# Clinical significance of polymorphisms of genes encoding collagen (*COL1A1, COL5A1*) and their correlation with joint laxity and recurrent patellar dislocation in adolescents

**DOI:** 10.1038/s41598-023-49378-6

**Published:** 2023-12-15

**Authors:** Krzysztof Małecki, Anna Fabiś-Strobin, Kinga Sałacińska, Katarzyna Kwas, Wojciech Stelmach, Jacek Beczkowski, Kryspin Niedzielski, Agnieszka Gach

**Affiliations:** Polish Mother’s Memorial Hospital Research Institute, Rzgowska 281/289, 93-338 Lodz, Poland

**Keywords:** Diseases, Medical research, Risk factors

## Abstract

The aim of this study was to assess the coexistence of polymorphisms of the *COL1A1* and *COL5A1* genes with clinically diagnosed laxity and the occurrence of recurrent patellar dislocation in adolescents. The research group comprised 50 cases of recurrent patellar dislocation. The mean age at diagnosis was 14.2 years (10–17, SD 2.6). The control group consisted of 199 participants without a diagnosis of recurrent patellar dislocation, with a mean age of 15.2 (10–17 years, SD 2.7). Joint laxity by the Beighton scale was assessed. Analysis of the allele distribution of the analysed genes *COL1A1* and *COL5A1* revealed no statistically significant difference between the study group and the control group (*p* = 0.859 and *p* = 0.205, respectively). Analysis of the Beighton score showed a statistically significantly higher result in the study group than in the control group (*p* < 0.001). No correlation between the presence of polymorphisms and joint laxity diagnosis was confirmed. In conclusion, *COL1A1* and *COL5A1* gene polymorphisms are not significantly more common in adolescents with recurrent patellar dislocation than in healthy peers; there is also no correlation between joint laxity and polymorphisms of the *COL1A1* and *COL5A1* genes.

Registered on ClinicalTrials.gov with ID: PMMHRI-2021.2/1/7-GW.

## Introduction

Ligamentous laxity is becoming both a clinically and scientifically important problem in medicine. Symptoms of joint laxity can vary in severity and indicate different clinical problems^1,2^. From a clinical perspective, this is of interest to mainly orthopaedic and trauma surgeons, physiotherapists and geneticists^1,3–5^.

The development of knowledge about this topic is still ongoing. Thanks to advances in genetic analysis, we can easily detect the presence of polymorphisms and analyse those present in cases with clinical features of laxity or diseases accompanied by laxity^4,6,7^. Due to the increasing number of musculoskeletal injuries in children and adolescents, interest in the relationship between laxity and musculoskeletal injuries is growing, which is noticeable in the corresponding rise in the number of publications on the subject. What is lacking thus far, however, is any semblance of systematization of knowledge regarding associating joint laxity severity with genotype characteristics, types of injuries, the associated risks, and an individual’s predisposition to them. A typical condition that orthopaedic surgeons must address in their daily practice is patellar instability. It is well known that patellar instability may manifest as first-time lateral dislocation or recurrent or habitual dislocation. It is widely believed that recurrent and habitual patellar dislocation are highly correlated with risk factors, of which ligamentous laxity ranks highest^8,9^.

Based on the literature and clinical experience, there is no doubt that recurrent and habitual patellar dislocation requires surgical treatment^10,11^. However, the consensus regarding the nonsurgical treatment of cases with first-time patellar dislocation is being challenged increasingly, often due to their diverse clinical presentation. According to the literature, the risk of redislocation is estimated to be between 20 and 70%^12,13^. Such dispersion is associated with the coexistence of many risk factors, among which joint laxity is considered to be one of the most important. If we could conduct a combined clinical and genetic assessment of joint laxity, we would be able to obtain a much more accurate assessment of the risk of recurrent patellar dislocation. When equipped with such knowledge, it would be easier to make decisions about surgical treatment after the first dislocation, avoiding possible complications such as osteochondral fractures and chondromalacia associated with subsequent dislocations and making patients more comfortable and returning them to full and safe physical activity. The treatment of patellar instability is ultimately about minimizing degenerative changes in the future but also about enabling the child to function properly, including in physical activity.

However, despite the many studies that have already been conducted, the genetic background of laxity and the factors causing it remain undetermined^14,15^.

As stated in Bell’s research, joint laxity is associated with particular single nucleotide polymorphisms (SNPs) of genes coding collagen, especially genes *COL1A1* and *COL5A1*^4^. As these are the genes that code collagen types I and V, they have an enormous impact on ligament conformation. It was determined that the rs1800012 polymorphism of *COL1A1* and the rs12722 polymorphism of *COL5A1* are involved in knee laxity (genu recurvatum) and may be related to general joint laxity (GJL) in females. These findings might also be relevant for recurrent patellar dislocation, which has not yet been studied.

Therefore, the aim of this study was to assess the coexistence of polymorphisms of the *COL1A1* and *COL5A1* genes with clinically diagnosed laxity and the occurrence of recurrent patellar dislocation in adolescents. The overriding goal is to create an algorithm for managing children and adolescents after the first patellar dislocation to minimize the risk of recurrent dislocation, improve the comfort of life and enable a quick return to amateur or professional sports. Adopting an appropriate approach to cases of first-time patellar dislocation has an important impact on reducing the occurrence of secondary degenerative changes and disability in adulthood. The work is also a response to the suggestion of Beckley et al. to test rs12722 *COL51A* in the population of patients with clinically diagnosed generalized joint laxity^6^.

### Research hypotheses

- *COL1A1* and *COL5A1* gene polymorphisms are significantly more common in adolescents with recurrent patellar dislocation than in healthy peers.
- There is a correlation between the occurrence of polymorphisms with clinically observed general joint laxity.

## Material and methods

The research group comprised 50 adolescents diagnosed with recurrent patellar dislocation, i.e., at least twice. The mean age at diagnosis was 14.2 years (10–17, SD 2.6), with 28 women and 22 men. The inclusion criteria for the study group were as follows: diagnosis of recurrent patellar dislocation, age between 10 and 17 years at the time of diagnosis, assessment of joint laxity at the time of diagnosis, and collected genetic material in the form of peripheral blood or buccal swabs. Criteria for exclusion from the study group were as follows: age over 18 at the time of diagnosis, one-time patellar dislocation, habitual patellar dislocation, permanent patellar dislocation, genetically determined syndromes including joint laxity (Down syndrome, Elhlers-Danlos syndrome, etc.), no completed research protocol and absence of consent to participate in the study.

The control group consisted of 199 adolescents (75 women and 124 men) without a diagnosis of recurrent patellar dislocation in whom 1) genetic material was collected from peripheral blood or in the form of a buccal swab and 2) the joint laxity of the Beighton scale was assessed^16^. The mean age in the control group was 15.2 (10–17 years, SD 2.7). The participants from the control group were recruited from those admitted to the Department of Orthopaedics and Traumatology for orthopaedic treatment not related to patellar instability or other injury. They were assessed by examiners in terms of meeting the inclusion and exclusion criteria. The inclusion criteria in the control group were as follows: age between 10 and 17 years, no history of joint injury or eligibility for assessment according to the Beighton scale, genetic material collected in the form of a buccal swab or peripheral blood. Exclusion criteria were as follows: history of musculoskeletal injury, age over 18 years, diagnosed congenital syndrome with features of joint laxity, absence of consent to participate in the study, and no completed research protocol.

On the day of qualification for the study, each patient in the study group and the control group was examined for clinical features of joint laxity according to the Beighton scale. It is a nine-point scale requiring 4 bilateral passive movements, with one as positive when hyperextension of the fifth finger in MCP exceeds 90°, hyperextension of the elbow and knee joint exceeds 10°, and the thumb can reach the volar part of the forearm, and 1 unilateral active movement of forward trunk flexion with straight knees and palms resting flat on the floor. The score ranges from 0 to 9 points, with one point for all nine tests. A Beighton score ≥ 4 is defined as joint laxity^16^. Oral epithelial material or peripheral blood cells were collected from each participant from the study and control groups for further genetic analysis. Both methods provided sufficient DNA for analysis. Blood was collected during routine preparation for surgery, and a buccal swab was taken from those who had no other indications for peripheral blood collection.

Genomic DNA was automatically isolated from peripheral blood leucocytes using the MagCore DNA Whole Blood Kit (RBC Bioscience, Taiwan). For buccal swabs, the manual Sherlock AX kit (A&A Biotechnology, Poland) was used. The distribution of genotypes for selected single nucleotide variants was determined by PCR–RFLP (polymerase chain reaction—restriction fragment length polymorphism). The c.104-441G > T variant of the *COL1A1* gene (rs1800012) was identified by a 693 bp fragment using the following primer pair: forward 5′-CCTGCTGACCGATGCTGA-3′ and reverse 5′-TGGCTTCCAACTCCAACCTC-3′. The PCR product was digested with Van91I restriction enzyme (Thermo Fisher, USA) and analysed on a 3% agarose gel. The presence of an intact 693 bp fragment indicated the mutated T allele, while the presence of two fragments (206 and 487 bp) indicated a normal G allele.

The c. *267C > T variant of the *COL5A1* gene (rs12722) was identified by using a 699-bp fragment using the primer pair: forward 5′-TTTGGATTTGAAGTGGGGCC-3′ and reverse 5′-CGTGGGACTGAGACTGGG-3′. The PCR product was digested with Bsh1236I restriction enzyme (Thermo Fisher, USA) and analysed on a 3% agarose gel. The presence of an intact 699 bp fragment indicated a mutated T allele, while the presence of two fragments (307 and 392 bp) indicated a normal C allele.

The Beighton scores obtained from the two groups (both study and control) were compared. The distribution of rs1800012 *COL1A1* (G/T) and rs12722 *COL5A1* (T/C) polymorphisms in both groups was assessed and compared. The results of the genetic analysis were related to the assessment of laxity in the Beighton scale, both in terms of quantity (score alone) and qualitatively (Beighton score ≥ 4). The relationship between the occurrence of joint laxity and polymorphisms among all patients (research and control groups together) was also assessed.

Adequate sample size prediction was performed prior to the study with the assumption of a statistical power minimum of 0.8. Due to the different distribution of alleles resulting in small samples in some tests, the statistical power (SP) was less than 0.8. It is presented in the following section after each *p* value. The statistical analysis was performed using the t test and the Mann‒Whitney U test for independent data. The independent Chi-square test was used for categorical data with Yates correction for a four-field array. Statistical significance was assumed for *p* < 0.05. The analysis was performed using the STATISTICA software package, ver. 10 (StatSoft, Inc. 2011, DASS).

The study was approved by the institutional review board of the Polish Mother’s Memorial Hospital (registration No 70/2021 and 89/2022) and registered on ClinicalTrials.gov with ID: PMMHRI-2021.2/1/7-GW. The study was carried out in accordance with the World Medical Association Declaration of Helsinki. Informed consent was obtained from all subjects and their parents or legal guardians.

The study was supported by a subsidy from the Ministry of Education and Science (ME&S) granted to Polish Mother’s Memorial Hospital-Research Institute (PMMH-RI), Grant No. 2021.2/1/7-GW.

### Informed consent

Informed consent was obtained from all subjects and their parents or legal guardians.

## Results

As a result of the analysis of the allele distribution of the analysed genes *COL1A1* and *COL5A1*, no statistically significant difference was found between the study group and the control group (*p* = 0.859 and *p* = 0.205, respectively, SP 0.82, Table 1). The observed frequencies of the mutated T allele of *COL1A1* in the study and control groups were 13% and 15%, respectively. Regarding the *COL5A1* gene polymorphism (rs12722), the observed distribution of altered T alleles in the study and control groups was 62% and 53%, respectively.

**Table 1:** Results of analysis of the distribution of the allele in both the recurrent patellar dislocation group and the control group.

Genotype	Study group		Control group	
	Number	%	Number	%
rs1800012				
GG	38	76	143	72
GT	11	22	52	26
TT	1	2	4	2
	Chi^2^ = 0.3036; *p* = 0.859			
rs12722				
CC	9	18	44	22
CT	20	40	98	49
TT	21	42	57	29
	Chi^2^ = 3.170; *p* = 0.205			

The analysis of the Beighton score showed a statistically significantly higher result in the study group than in the control group (*p* < 0.001 with SP 0.89, Table 2). This difference was also statistically significant for both women and men (*p* = 0.004 with SP 0.7 and *p* = 0.031 with SP 0.41, respectively, Table 3).

**Table 2:** Results of analysis of the difference in both groups: recurrent patellar dislocation group (study group) and control group.

	No.	Beighton-scale points			
		Mean (range)	SD	z	*p* value
Study group	50	3.16 (0–9)	2.06	3.804	*p* < 0.001
Control group	199	2.05 (0–8)	1.79		

*SD* standard deviation.

**Table 3:** Results of analysis of the difference in both groups: recurrent patellar dislocation group (study group) and control group according to sex.

	Beighton-scale points									
	Women					Men				
	No.	Mean (range)	SD	z	*p* value	n	Mean (range)	SD	z	*p* value
Study group	28	3.68 (0–9)	2.09	2.696	*p* = 0.004	22	2.50 (0–6)	1.87	1.882	*p* = 0.031
Control group	75	2.56 (0–7)	1.79			124	1.73 (0–8)	1.74		

*SD* standard deviation.

The women had significantly higher Beighton scores than the men in both the study and control groups (*p* = 0.022 with SP 0.65 and *p* < 0.001 with SP 0.89, respectively, Table 4).

**Table 4:** Results of analysis of the difference between sexes in each group: recurrent patellar dislocation group (study group) and control group.

	Beighton-scale points									
	Study group					Control group				
	No.	Mean (range)	SD	z	*p* value	n	Mean (range)	SD	z	*p* value
Women	28	3.68 (0–9)	2.09	2.071	*p* = 0.022	75	2.56 (0–7)	1.79	3.213	*p* < 0.001
Men	22	2.50 (0–6)	1.87			124	1.73 (0–8)	1.74		

*SD* standard deviation.

No significantly higher Beighton scores were detected in cases with polymorphisms (*p* = 0.370 with SP 0.11 for rs1800012 and *p* = 0.358 with SP 0.78 for rs12722, Table 5).

**Table 5:** Results of analysis of the difference between Beighton score depending on the presence of polymorphisms in all study and control groups together.

	No.	Beighton-scale points			
		Mean (range)	SD	z	*p* value
rs1800012 GG	181	2.25 (0–9)	1.92	0.332	*p* = 0.370
GT + TT	68	2.11 (0–7)	1.79		
rs12722 CC	53	3.24 (0–9)	2.14	0.365	*p* = 0.358
CT + TT	196	2.30 (0–9)	1.89		

*SD* standard deviation.

In the qualitative analysis, considering 4 or more points on the Beighton scale as joint laxity, no statistically significant differences were found in the analysis of the occurrence of polymorphisms for both genes depending on present laxity (SP 0.93, Table 6). A similar result was obtained in the case of the distribution of alleles (SP 0.91, Table 7). This indicated that there was no correlation between the presence of polymorphisms and the diagnosed joint laxity.

**Table 6:** Results of analysis of general laxity distribution depending on the occurrence of polymorphisms in a study and control group together.

	Beighton score ≥ 4 No.	Beighton score < 4 No.
rs1800012		
GG	48	133
GT + TT	17	51
	Chi^2^ = 0.0592; *p* = 0.808	
rs12722		
CC	40	13
CT + TT	144	52
	Chi^2^ = 0.014; *p* = 0.906	

Beighton score ≥ 4 means general laxity presence.

**Table 7:** Results of the analysis of general laxity distribution depending on the distribution of alleles in a study and control group together.

	Beighton score ≥ 4 No.	Beighton score < 4 No.
rs1800012		
GG	48	133
GT	16	47
TT	1	4
	Chi^2^ = 0.129; *p* = 0.937	
rs12722		
CC	40	13
CT	89	29
TT	55	23
	Chi^2^ = 0.674; *p* = 0.714	

Beighton score ≥ 4 means general laxity presence.

## Discussion

In the assessment of the distribution of alleles of the analysed genes *COL1A1* and *COL5A1*, there was no link between the existence of the polymorphism and the occurrence of recurrent patellar dislocation, both in relation to the polymorphism in one allele and in both alleles. The frequency of mutated alleles for *COL1A1* and *COL5A1* gene polymorphisms that have been observed is similar to the distribution reported in population databases. In the European subpopulation, the frequency of the mutated T allele for rs180012 ranges between 18 and 19%, while the altered T variant of the rs12722 polymorphism is estimated at 56–59% (databases: ALFA Allele Frequency, gnomAD genomes, 1000 Genomes).

There was also no relationship between the presence of polymorphisms in the *COL1A1* and *COL5A1* genes and the occurrence of joint laxity in quantitative and qualitative terms. A significantly higher Beighton score was found in the group of adolescents with recurrent patellar dislocation than in the control group. Similarly, laxity occurrence described qualitatively (Beighton score ≥ 4) was more frequent in the study group. These results confirm current thinking supporting the view that recurrent patellar dislocation is associated with ligamentous laxity. However, the study did not confirm the relationship between laxity and polymorphisms within the *COL1A1* and *COL5A1* genes. What has become apparent is that the joint laxity in these cases is due to more complex problems related to gene expression, disorders of collagen synthesis, and its matrix.

In addition, an interesting observation is the fact that joint laxity refers more often to women than men, which confirms that this is not the only risk factor for patellar instability.

Much work remains to be done to understand the nature of ligamentous laxity and its relationship with genotype, expression, and clinical appearance. The study should be extended to include the analysis of polymorphisms in other genes encoding collagen, e.g., *COL11A1*, *COL11A2*, analysis of gene expression, processes of extracellular matrix formation, and other factors influencing the formation of connective tissue.

There is no reference of the results of this study to data from the literature because it is the first study of this character evaluating a homogenous group of adolescents with recurrent patellar dislocation. The available literature provides us with a limited number of similar studies, to which these results can only be partially related.

In the available literature, there are several studies assessing the genetic or molecular cause of ligamentous laxity expressed with knee joint laxity and in Ehlers‒Danlos syndrome (EDS). Beckley assessed the coexistence of *COL5A1* and *COL11A2* polymorphisms with hypermobility within the knee joint. Their research work revealed decreased external knee rotation in a group with variant CC of *COL5A1* rs12722, but there were no such differences found in the analysis in terms of genu recurvatum or anterior–posterior tibial translation^6^.

Research carried out by Tuna et al. assessed the relationship between serum lithium, zinc, and strontium levels and EDS expression-related genes. Significantly reduced levels were found in a group of women with generalized joint laxity (Beighton score ≥ 4). The expression of *COL1A1, COL1A2,* and *COL5A1* was lower in this group^14^.

Alanis-Funes et al., whose diagnosis is based mainly on clinical features, emphasize the lack of research on the differentiation of general ligamentous laxity disorders from hypermobile EDS. Researchers performed whole-exome sequencing in 5 participants with general joint hypermobility, finding mutations of three genes, *MUC3A*, *RHBG* and *ZNF717,* in all cases studied^7^.

*MUC3A* variants are mentioned as they are associated with inflammatory bowel disease, and three out of five patients from the study presented gastrointestinal problems. This may have some clinical and genetic relevance that should be taken into account in further analyses when dealing with such cases.

Gensemer et al. focused on genetic, epidemiologic, and pathogenetic findings in the hypermobile type of EDS, underlining multifactorial causes of joint hypermobility including not only genes related to collagen synthesis but also other gene expressions and collagen molecular biology. The authors concluded that the unknown aetiology and pathogenesis of the hypermobility condition should encourage scientists to perform animal studies in collective research networks^15^.

We also have to take into consideration that collagen gene variants may also be associated with many other conditions, such as fibromuscular dysplasia, arteriopathy leading to arterial dissection and other developmental conditions, such as DDH^17,18^. This proves the importance of the genetic approach in finding aetiologic factors of the conditions we cope with in our practice. The ongoing development of the diagnostic methodology in genetics is making it more available and should lead physicians to use it more often.

Another scientific approach worth considering is the analysis of the familial occurrence of patellar instability. Genetic and molecular analysis in these cases may be crucial for finding the causes of laxity and predicting the recurrence of dislocation in first-time dislocators, thereby enabling implementation of more aggressive management in this group depending on the congenital risk factors. It may be that the search for common genome and expression features will prove simpler and facilitate finding the causes of laxity. The present study showed that the analysis of *COL1A1* and *COL5A1* polymorphisms is insufficient and that the analysis of other genes responsible for collagen synthesis, its regulation, and expression is required, highlighting a subject for further research.

## Conclusions

- *COL1A1* and *COL5A1* gene polymorphisms are not significantly more common in adolescents with recurrent patellar dislocation than in healthy peers
- There is no correlation between joint laxity and polymorphisms of the *COL1A1* and *COL5A1* genes
- Currently, the mere presence of clinically proven laxity should be considered an important factor in recurrent patellar dislocation
- There is still a need to identify the genetic and molecular cause of joint laxity affecting the occurrence of patellar instability

## Data Availability

Data supporting the findings of this study are available from the corresponding author [KM] upon reasonable request.
